# Prevalence and factors associated with smoking among university staff

**DOI:** 10.1192/j.eurpsy.2024.826

**Published:** 2024-08-27

**Authors:** I. Sellami, A. Feki, A. Abbes, M. Masmoudi, M. Hajjaji, K. Jmal Hammami

**Affiliations:** ^1^Occupational medicine, Hédi Chaker Hospital; ^2^ Medecine university; ^3^Rheumatoloy, Hédi Chaker Hospital, Sfax, Tunisia

## Abstract

**Introduction:**

Smoking among university professionals could influence student smoking behavior, making it important to understand the associated factors to prevent this phenomenon.

**Objectives:**

To determine the prevalence of smoking among University staff in Sfax, Tunisia, and identify its associated factors.

**Methods:**

We conducted a cross-sectional survey using a self-administered questionnaire distributed to 100 university staff. The questionnaire included socio-professional characteristics, assessment of physical workload using the Borg CR-10 scale, and evaluation of nicotine dependence using the Fagerström test.

**Results:**

Our study included 62 participants, with 67.7% of them being women. Active smoking was reported by 50% of male participants. We observed symptoms of severe to very severe depression, anxiety, and stress in 6.4%, 22.5%, and 9.7% of our participants, respectively. Nicotine dependence, as assessed by the Fagerström test, was high to very high in half of the smokers. Bivariate analysis indicated a significant association between smoking and male gender, perceived workload (33.9%), and body mass index.

**Conclusions:**

Smoking among university staff is a prevalent phenomenon, especially among male participants. The association of smoking with perceived workload suggests the need for preventive measures to reduce these physical constraints. It is paramount to take actions to encourage smoking cessation among university staff.

**Disclosure of Interest:**

None Declared

